# Draft Genome Sequence of Cellulophaga sp. E6, a Marine Algal Epibiont That Produces a Quorum-Sensing Inhibitory Compound Active against Pseudomonas aeruginosa

**DOI:** 10.1128/genomeA.01565-14

**Published:** 2015-02-12

**Authors:** J. E. Lafleur, S. K. Costa, A. S. Bitzer, M. W. Silby

**Affiliations:** a Department of Biology, University of Massachusetts Dartmouth, North Dartmouth, Massachusetts, USA; b Department of Emergency Medicine, Alpert School of Medicine of Brown University, Providence, Rhode Island, USA

## Abstract

The genus Cellulophaga is composed of obligate aerobic Gram-negative bacteria commonly found in association with marine algae. We report the approximately 4.42-Mbp draft genome sequence of Cellulophaga sp. E6, which inhibits *N*-(3-oxododecanoyl)-l-homoserine lactone (3-oxo-C_12_-HSL)–mediated quorum sensing (QS), *lasB* transcription, and biofilm formation by Pseudomonas aeruginosa.

## GENOME ANNOUNCEMENT

We isolated Cellulophaga sp. E6 (1, 2) from the surface of a coastal seaweed as part of a bioprospecting search for marine bacteria which have the potential to control P. aeruginosa virulence through inhibiting acylhomoserine lactone (AHL)-based quorum sensing (QS). Cellulophaga spp. are obligate aerobes which hydrolyze agar and carrageenan. Most Cellulophaga spp. display gliding motility (3, 4), and are strongly proteolytic (5), and like many *Flavobacteriaceae*, Cellulophaga can be algalytic (6–9). Algalysis is achieved both through direct attack, and through indirect chemical means (8). Recent reports have focused on potential applications for novel enzymes from Cellulophaga species that break down carrageenan (3, 10, 11).

Using a luciferase-based reporter construct (P_lasB_-*luxCDABE*) specific for 3-oxo-C_12_-HSL (12), we have shown that Cellulophaga sp. E6 inhibits 3-oxo-C_12_-HSL QS in P. aeruginosa which uses this system and another AHL-based QS system (C4-HSL) to control virulence gene expression and biofilm formation (13). Supernatant from Cellulophaga sp. E6 culture reduced expression of the 3-oxo-C_12_-HSL-dependent virulence-associated gene *lasB*, and reduced biofilm formation in a dose-dependent manner. Activity-guided purification of the QS inhibitory activity has shown that the target molecule is smaller than 1000 Da, water-soluble, and stable to temperatures of 50°C.

Genomic DNA was isolated from Cellulophaga E6 using the Wizard Genomic DNA purification kit (Promega). The sequencing library was prepared using Nextera XT sequencing library preparation kit (Illumina). Sequencing was carried out using a MiSeq Genome Sequencer (Illumina) at Tufts University Genomics Core, which generated 4,425,292 2 × 250 bp paired-end reads. *de novo* assembly was done using CLC Genomics Workbench v7.0.4 (CLC Bio, Denmark) with the minimum contig size set to 250 bp, resulting in 72 contigs. The contigs range in size from 528 to 692,305 bp. The assembled genome had 300-fold coverage, with an *N*_50_ scaffold size of 203,379 bp. Annotation was added by the NCBI Prokaryotic Genome Annotation Pipeline. The total size for the combined contigs is 4,420,065 bp, leading us to estimate the genome as approximately 4.42 Mb; it is predicted to contain 3,630 protein-coding genes, 36 tRNA genes, and 2 rRNA genes. 16S and 23S rRNA genes were detected, but since the assembly is based on short reads, the numbers and locations of multiple copies could not be determined. The DNA G+C content was 34.6%, which is consistent with those of other Cellulophaga genomes.

Some Cellulophaga spp. have been reported to quorum sense with AI-2 (8). A potential mechanism for Cellulophaga spp. to inhibit P. aeruginosa QS is via 4-hydroxy-2,5-dimethyl-3(2H)-furanone (HDMF), a by-product of *in vitro* LuxS-mediated synthesis of the QS molecule AI-2 (4,5-dihydroxy-2,3-pentanedione) (14, 15), which has been reported to inhibit P. aeruginosa QS, and biofilm formation (16). However, we have not detected sequences related to *luxS* in the Cellulophaga sp. E6 genome, suggesting that Cellulophaga sp. E6 does not employ AI-2 QS and thus may inhibit QS using a novel small molecule. The genome sequence of Cellulophaga sp. E6 will facilitate the search for the genetic basis of the QS inhibiting molecule, complementing the biochemical analysis of its synthesis.

### Nucleotide sequence accession numbers.

This whole-genome shotgun project has been deposited at DDBJ/EMBL/GenBank under the accession no. JQCT00000000. The version described in this paper is version JQCT01000000.
